# Physical activity level and its determinants in patients with cancer: a secondary analysis using a decision-tree approach

**DOI:** 10.1007/s11764-026-02055-9

**Published:** 2026-06-08

**Authors:** Alice Avancini, Diana Giannarelli, Anita Borsati, Christian Ciurnelli, Michele Milella, Sara Pilotto, Federico Schena

**Affiliations:** 1 Department of Neurosciences, Biomedicine and Movement Science, University of Verona, P.Le L.A. Scuro 10, 37134 Verona, Italy; 2 Section of Innovation Biomedicine - Oncology Area, Department of Engineering for Innovation Medicine (DIMI), University of Verona, and University and Hospital Trust (AOUI) of Verona, Verona, Italy; 3 Facility of Epidemiology and Biostatistics, G-STeP, Fondazione Policlinico Universitario A. Gemelli, IRCCS, Rome, Italy

**Keywords:** Physical activity, Patients with cancer, Decision tree, Determinants, Aerobic exercise, Resistance training

## Abstract

**Purpose:**

Low physical activity is associated with poorer outcomes in patients with cancer. Understanding the determinants of physical activity is essential to designing targeted interventions. This study aimed to identify the determinants of total, aerobic, and resistance physical activity among patients with cancer using a decision-tree machine learning approach.

**Methods:**

This secondary analysis pooled data from three harmonized cross-sectional studies conducted between 2018 and 2024, including adult patients (aged ≥ 18 years) with any cancer type and stage. Physical activity was assessed through validated self-report questionnaires and classified according to current guidelines. Sociodemographic and clinical variables were considered as potential correlates. Decision-tree models using the Classification and Regression Trees method were applied to identify predictors of adherence to aerobic and resistance exercise recommendations.

**Results:**

A total of 1132 patients were included. Only 17.5% met aerobic activity recommendations (i.e., 150 min/week or ACSM guidelines), and 12.7% met resistance training recommendations (i.e., 2 times/week); 2.6% met both. cancer site emerged as the primary determinant of both aerobic and resistance activity. Occupational status, body mass index, and educational level further influenced adherence patterns. Neither cancer stage nor treatment timing significantly predicted physical activity behavior. Distinct determinants were identified for aerobic versus resistance exercise.

**Conclusions:**

Among the factors included in the decision-tree models, physical activity was mainly influenced by cancer site and sociodemographic characteristics. Aerobic and resistance activities are governed by different predictors, underscoring the need for modality-specific assessment and intervention strategies.

**Implications for Cancer Survivors:**

Tailored, cancer-specific supports that account for sociodemographic context and address barriers to both aerobic and resistance exercise are urgently needed to increase participation in physical activity and optimize survivorship outcomes.

## Introduction

Large longitudinal studies have consistently linked lower physical activity with poorer survival among patients with cancer [1, 2]. In this context, a meta-analysis of 136 studies found that high levels of physical activity reduce all-cause mortality by 37%, with the greatest benefits observed for breast and colon cancers [1]. Decreased mortality rates in patients with cancer are observed at physical activity levels of approximately 150 min of moderate-intensity activity per week, corresponding to 10–15 MET-hours/week [1]. Moreover, features directly modifiable by regular physical activity, such as cardiorespiratory fitness, muscle strength, and mass, are independent prognostic factors in cancer [3]. To support this, a recent phase III trial on 889 patients with colon cancer after chemotherapy demonstrated that 3 years of structured exercise lowered the recurrence and mortality risk by 28% and 37%, respectively, confirming the therapeutic potential of physical activity [4]. Beyond mortality, exercise improves both physical and psychological aspects, as well as symptom/treatment side effects, thereby enhancing their overall quality of life [5]. Building on this evidence, most available guidelines recommend that patients with cancer engage in at least 150 min of moderate-intensity or 75 min of vigorous-intensity aerobic activity per week, combined with resistance training on two or more days [6].

However, after a cancer diagnosis, patients usually tend to decrease their physical activity, and as a result, most of them are insufficiently active [7, 8]. Understanding the key factors that influence patients’ physical activity is fundamental for developing interventions specifically targeted at those at higher risk of inactivity [7, 8]. Several prior studies have identified multiple factors, both disease-related (e.g., type of treatment, severity) and unrelated to cancer (e.g., age, educational level), that may contribute to physical activity behavior [8–10]. Traditionally, these analyses have employed the classical regression models. Although informative, these approaches are typically knowledge-driven, assume linear and independent effects, and may overlook complex or nonlinear relationships that characterize behaviors, such as physical activity [11–13]. On the other hand, machine learning approaches offer a data-driven framework capable of handling complex datasets with fewer assumptions [14, 15]. Among these, decision trees have attracted particular interest due to their ability to identify the most relevant predictors, to flexibly model nonlinear relationships, and to detect multiple interactions without prior specification [16]. Decision tree models have highly interpretable outputs in the form of hierarchical, tree-like structures, which not only enhance transparency but also facilitate interpretation [17].

Although machine learning has been used to predict adherence to physical activity guidelines in adults [18–20], few studies have applied it to cancer populations. Recently, Ma et al. developed a machine learning prediction model for patients with lung cancer, identifying 15 sociodemographic, clinical, and psychophysical predictors of physical activity adherence, with random forests achieving the best accuracy [21].

However, to our knowledge, no other studies have employed a decision-tree-based approach to examine the determinants of physical activity in patients with cancer. This approach would help to elucidate whether disease type, characteristics, and treatments, beyond the already recognized sociodemographic factors, may also play a significant role. Additionally, no studies have yet examined whether distinct determinants influence both aerobic and resistance exercises. Therefore, this study aimed to identify the main determinants of aerobic and resistance physical activity in patients with cancer using a decision-tree approach. Rather than testing an intervention to improve adherence, this analysis was designed to generate a clinically accessible risk-stratification framework capable of identifying subgroups of patients who may be more likely to fall below current physical activity recommendations.

## Methods

### Study design and participants

This study is a secondary analysis pooling data from three cross-sectional studies. Data on physical activity, sociodemographic characteristics, and clinical information was extracted from the self-reported questionnaires. All investigations shared similar methodological frameworks and were coordinated by the same research group between 2018 and 2024. The three studies have similar eligibility criteria, including adult patients (i.e., aged ≥ 18 years) with a confirmed diagnosis of cancer, any type and any stage. All studies received approval from the Ethics Committee (protocol numbers 49018, 48647, and 54507). All procedures were conducted in accordance with the Declaration of Helsinki, the Oviedo Declaration, and Good Clinical Practice principles. The manuscript adheres to the Strengthening the Reporting of Observational Studies in Epidemiology (STROBE) recommendations.

### Assessment of physical activity

Physical activity was assessed using validated self-report questionnaires, harmonized across the three parent studies. The aerobic activities were evaluated using the Godin-Shepard Leisure-Time Exercise Questionnaire (GLTEQ), evaluating the weekly frequency of vigorous, moderate, and light-intensity exercise, in two studies [7, 9]. By calculating the frequency for the corresponding MET, i.e., (vigorous × 9) + (moderate × 5), a Leisure Score Index (LSI) was obtained. Patients achieving an LSI ≥ 24 were classified as sufficiently active for aerobic activity, corresponding to at least 150 min of moderate-intensity or 75 min of vigorous-intensity activity per week [22]. In the other study, aerobic exercise was assessed using an open-ended question adapted by Schmitz and colleagues [23], asking about the frequency and duration of activity at least at moderate intensity during the past week [24]. Patients reporting at least 150 min per week of physical activity were considered to meet the recommendation for aerobic exercise [6]. Strength activities were assessed in two studies (total analytic sample of *n* = 684) [7, 24], using an open-ended question from Schmitz and colleagues [23], which asked about the frequency of such activities in the last week. Participants who performed two or more weekly sessions of strength training were considered adherent to the current recommendations for resistance activity [6]. Patients meeting both recommendations for aerobic and strength training were considered sufficiently active, according to the current guidelines [6].

### Correlates

Potential correlates of physical activity, i.e., self-reported sociodemographic and clinical information, were assessed with the same questions. Sociodemographic variables included gender, age, body mass index (BMI), educational level, marital status, occupational status, and perceived income adequacy. Missing data were recorded for each variable category. Clinical variables were also collected through closed-ended questions and included cancer site, cancer stage, time since diagnosis, and timing of therapy. All variables were harmonized before merging the datasets to ensure methodological consistency.

### Statistical analysis

Data were summarized as absolute counts and percentages for categorical variables and as medians and interquartile ranges (IQRs) for quantitative variables. The two patient groups (insufficiently active versus sufficiently active) were compared using the Mann–Whitney test or the chi-square test, as appropriate. A decision-tree analysis was used to predict a binary outcome from patients’ characteristics. Cancer site was entered into the decision-tree models as an unweighted categorical variable; no weighting was applied according to the number of participants within each cancer-site category. This model works by recursively splitting data into subsets, and each terminal node represents the final classification outcome. Splitting was performed using the Classification and Regression Trees (CRT) method, which maximizes within-node homogeneity by minimizing impurity (non-homogeneity within the node), measured by the Gini index. Improvement changes are reported at each step, with a default value of 0.0001; splitting stops when this value is reached. Analyses were performed using IBM SPSS statistical software, version 28.0.

## Results

### Participants characteristics

A total of 1132 patients were included in this analysis. Relevant characteristics of the sample are presented in Table 1. Overall, data from 1048 and 684 patients were available for aerobic and resistance training, respectively. Among these patients, 17.5% and 12.7% met the recommendations for aerobic and resistance activities, respectively. Among the 684 patients with data available for both types of physical activity, 2.6% met both recommendations. This low number of physically active patients precluded performing decision-tree analysis.

**Table 1:** Socio-demographic characteristics of the study participants

Variable	*N* = 1132
Gender, *N* (%)	
Female	616 (54.4)
Male	516 (45.6)
Age (median, IQR)	63 (54–71)
Body mass index (median, IQR)	24.62 (22.0–27.7)
Education, *N* (%)	
Elementary	117 (10.3)
Secondary	352 (31.1)
High school degree	448 (39.6)
College/University	164 (14.5)
Postgraduate	39 (3.4)
Missing	12 (1.1)
Marital status, *N* (%)	
Single	136 (12.0)
Married	792 (70.0)
Divorced	96 (8.5)
Widowed	92 (8.1)
Missing	16 (1.4)
Occupational status, *N* (%)	
Full-time employed	328 (29.0)
Part-time employed	97 (8.6)
Seeking employment	21 (1.9)
Retired	542 (47.9)
Homemaker	128 (11.3)
Missing	16 (1.4)
Perceived income adequacy, *N* (%)	
More than adequate	224 (19.8)
Adequate	544 (48.1)
Barely adequate	277 (24.5)
Inadequate	65 (5.7)
Missing	22 (1.9)
Cancer site, *N* (%)	
Upper gastrointestinal tract	469 (41.4)
Breast	197 (17.4)
Lung	171 (15.1)
Colorectum	95 (8.4)
Urogenital	50 (4.4)
Other	131 (11.6)
Missing	19 (1.7)
Cancer stage, *N* (%)	
Early	193 (17.0)
Advanced	240 (21.2)
Metastatic	343 (30.3)
In remission/cured	177 (15.6)
Unknown	144 (12.7)
Missing	35 (3.1)
Months from diagnosis (median, IQR)*	12 (5–33)
Timing of therapy, *N* (%)	
About to start	43 (3.8)
Ongoing	893 (78.9)
Completed	120 (10.6)
Unknown	47 (4.2)
Missing	29 (2.6)
Physical activity level, *N* (%)	
Meeting the recommendations	19 (1.6)
Not meeting the recommendations	665 (58.7)
Missing	448 (39.6)
Aerobic physical activity level, *N* (%)	
Meeting the recommendations	180 (15.9)
Not meeting the recommendations	865 (76.4)
Missing	87 (7.7)
Resistance physical activity level, *N* (%)	
Meeting the recommendations	87 (7.7)
Not meeting the recommendations	597 (52.7)
Missing	448 (39.6)

*Missing for 435 patients

### Determinants of aerobic and resistance activity behavior

**Fig. 1:**
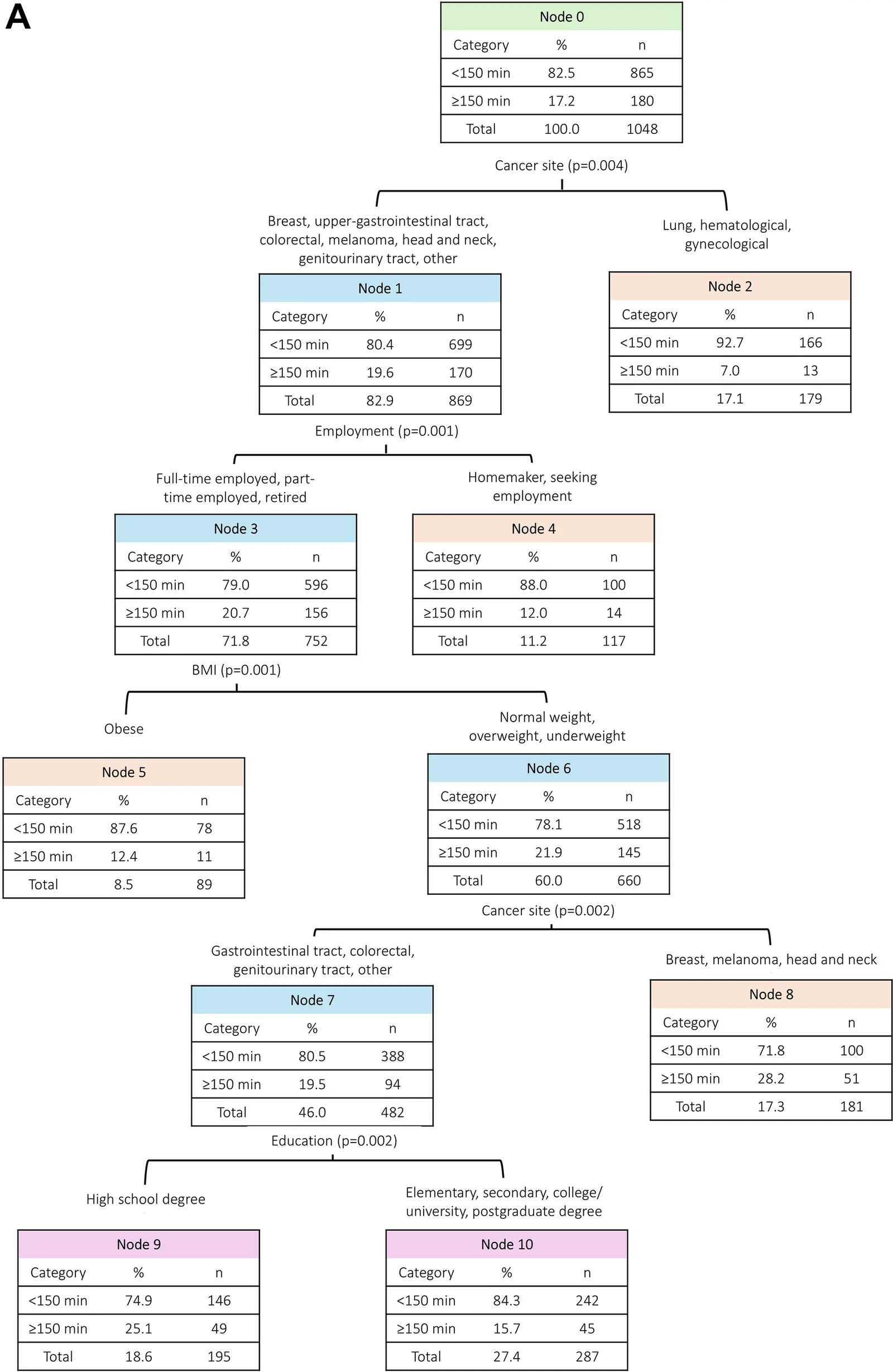

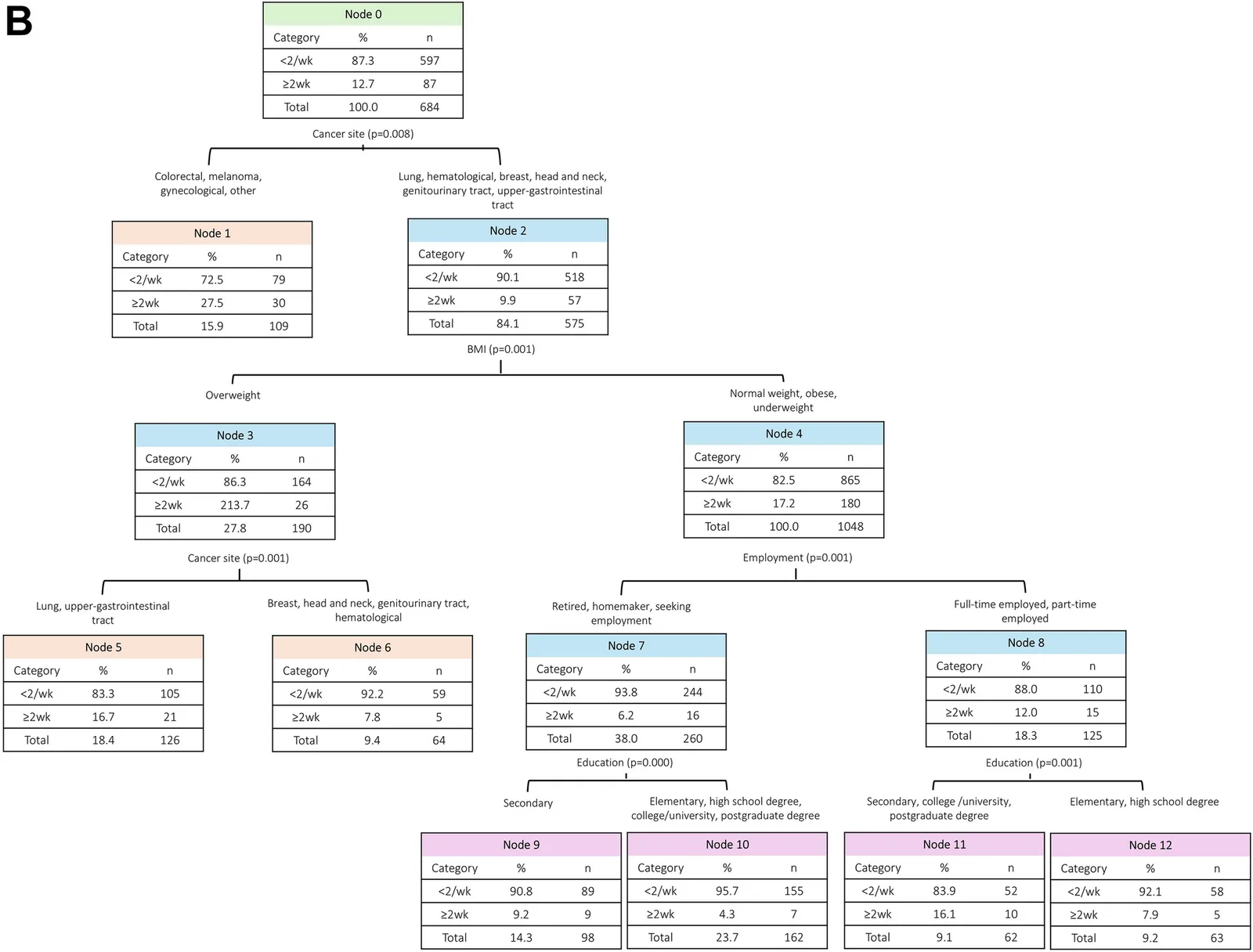
The decision-tree model analyzing factors predicting adherence to aerobic (**A**) and resistance (**B**) physical activity

Figure 1 displays the decision-tree model identifying predictors of adherence to aerobic [Fig. 1A] and resistance [Fig. 1B] activities.

Regarding aerobic activity, the cancer site was the first splitting variable (Node 0), yielding two main subgroups: patients with breast, genitourinary, upper gastrointestinal, colorectal, melanoma, or head-neck cancers (node 1), showing a higher adherence rate (19.6%), and those with lung, gynecological, or hematological malignancies (node 2), reporting lower levels (7.3%). For the second group, no further determinants were found. Within the first group, occupational status was the next most relevant predictor. Patients who were retired or employed, full-time or part-time, had a higher probability of meeting aerobic activity recommendations (node 3; 20.7%) than those who were homemakers or job seekers (node 4; 12.0%). Among employed participants in node 3, BMI category further differentiated adherence levels, with underweight, normal-weight, and overweight individuals (node 6) reporting higher levels of aerobic activity (21.9%) than obese individuals (node 5; 12.4%). Within node 6, a subsequent split by cancer site distinguished patients with genitourinary, upper gastrointestinal, or colorectal cancers (node 7; 19.5%) and those with breast, melanoma, or head-neck cancers (node 8; 28.2%). Finally, among patients in node 7, those with a university degree or higher (node 10) exhibited the highest adherence rate (24.7%), compared with those with lower educational levels (node 9; 15.1%).

Regarding resistance activities, the cancer site was the primary predictor of adherence. Patients with upper gastrointestinal tract, breast, head and neck, lung, genitourinary, or hematologic cancers (node 2) showed lower adherence (9.9%) than those with other cancers (node 1; 27.5%). Among the node 2 subgroup, BMI represented the next key determinant. Patients who were overweight (node 3) demonstrated higher adherence (13.7%) compared to those classified as normal weight, obese, or underweight (node 4; 8.1%). Within node 3, patients with upper gastrointestinal or thoracic cancers (node 5) were less active (16.7%) than those with breast, head and neck, genitourinary, or hematologic cancers (node 6; 7.8%). Within node 4, patients who were retired or unemployed (node 7) had lower adherence (6.2%) than those working full-time or part-time (node 8; 12.0%). In both groups, educational level further refined the probability of adherence, with patients with higher education (high school diploma or above) consistently more likely to engage in resistance training (nodes 9–12).

## Discussion

In this secondary analysis of three harmonized datasets of patients with cancer, we identified determinants of aerobic and resistance physical activity using a decision-tree approach. By translating sociodemographic and clinical characteristics into an interpretable classification structure, this approach may help clinicians identify patient profiles associated with lower adherence and guide individualized physical activity support. Overall, we found low adherence to recommended physical activity levels: only 17.5% and 12.7% of patients met the guidelines for aerobic and resistance training, respectively. Just 2.6% of patients achieved both recommendations. A prior systematic review, including 41 studies, found that, on average, 34.2% of patients met the physical activity guidelines [8]. This percentage is higher compared to that found in our study. Nevertheless, it should be noted that in such investigations, meeting the guidelines was defined as 150 min of moderate-intensity or 75 min of vigorous-intensity physical activity per week, without distinguishing between aerobic and resistance activities [8]. However, this criterion may have overestimated the percentage of sufficiently active patients since it also includes the resistance-training component. By contrast, our analysis applied separate thresholds for aerobic and resistance exercise, providing a more nuanced and stringent assessment of guideline adherence. Consequently, the lower prevalence observed in our study may reflect a more accurate estimate of proper compliance, highlighting a substantial gap between current recommendations and patients’ real-world physical activity behaviors. The “dark side” of this approach is that the low proportion of physically active patients found prevented the development of a reliable decision-tree model. On the other hand, it has permitted, for the first time, the analysis of the determinants of aerobic and resistance training separately.

In both models, the cancer site was the primary discriminator of activity levels. In this context, for aerobic activity, patients with lung, hematological, and gynecological cancers were more likely to exhibit lower adherence than patients with other cancer types. By contrast, patients with melanoma, colorectal, and gynecological cancers were more likely to engage in muscle-strengthening activities compared to others. To our knowledge, this is the first study to identify cancer site as a key determinant differentiating adherence patterns between aerobic and muscle-strengthening activities. Although the underlying reasons for these findings remain unclear, they could reflect differences in symptom burden, treatment intensity, functional limitations, and clinical recommendations across cancer types [7, 25, 26]. However, these findings may provide clinically relevant insights for exercise prescription in oncology. For instance, for cancer types commonly associated with substantial weight loss, sarcopenia, and muscle wasting (e.g., pancreatic and head and neck cancers), resistance training may play a more crucial role in preserving or restoring muscle mass and physical function [27]. Conversely, in cancers more frequently associated with excess body weight or adiposity (e.g., breast, prostate, and gynecological cancers), aerobic exercise may warrant greater emphasis to address cardiometabolic risk and weight management [27]. Therefore, knowing that specific cancer types are more likely to engage in either aerobic or resistance exercise could facilitate the development of more targeted, cancer-tailored exercise interventions that focus on the physical activity modality in which patients are least active and could stand to benefit most.

Interestingly, neither cancer stage nor treatment timing was a predictor in the decision trees. Despite clinical assumptions that advanced disease or ongoing treatment may limit physical activity, our models did not identify these variables as determinants of physical activity. This absence suggests that other factors, e.g., functional capacity, symptom profiles, and psychosocial conditions, could mediate the relationship between disease severity and physical activity. Unfortunately, these features were not captured in the present datasets but represent important aspects to be explored in future research. The finding is also consistent with emerging literature that shows high inter-individual variability within the same stage, challenging linear assumptions about disease progression and behavior [28].

Sociodemographic variables demonstrated substantial predictive value in both decision-tree models. Occupational status emerged as a significant determinant of aerobic and resistance activity, with patients who were employed showing higher adherence compared with retirees, homemakers, or those seeking employment. Occupational status may reflect differences in daily routines, functional capacity, social engagement, and socioeconomic resources, all of which may influence physical activity behavior.. In addition, employed individuals may perceive physical activity as more instrumental for sustaining work ability and independence. Interestingly, BMI showed differential associations with physical activity across exercise modalities. In the aerobic activity model, obesity was associated with lower levels of physical activity. It is consistent with prior literature and may reflect barriers such as reduced mobility, heightened fatigue perception, and lower exercise self-efficacy. In the resistance training model, being overweight was associated with higher participation rates than in other BMI categories. To our knowledge, this is the first study reporting this finding, which may be partly explained by physiological/functional factors. In particular, being overweight may reflect a more favorable balance between muscle and fat mass, potentially facilitating engagement in muscle-strengthening activities without the functional limitations often observed at higher BMI levels. Together, these findings suggest that BMI may influence engagement in physical activity in a modality-specific manner rather than acting as a uniform barrier across exercise types. The role of educational level as a determinant of aerobic and resistance activities was heterogeneous and difficult to interpret. While higher education is generally associated with higher physical activity levels, our finding likely reflects indirect effects mediated by broader socioeconomic and contextual factors rather than education alone, and thus warrants further investigation.

Limitations of this study include reliance on self-reported activity, which may be subject to misclassification. In addition, resistance-training and combined physical activity data were unavailable for 448 participants, corresponding to 39.6% of the total sample. This missingness mainly reflects the fact that resistance training was assessed in only two of the three parent studies and may limit the generalizability of findings related to resistance training and combined adherence to physical activity recommendations. Additionally, several potentially relevant predictors, including symptom burden, performance status, objective economic status (e.g., income), and treatment-related toxicity, were not available in the harmonized datasets; their absence may have contributed to the exclusion of stage or treatment characteristics from the decision-tree models. Finally, cancer-site categories were not equally represented in the sample, and this uneven distribution may have influenced the stability of cancer-site splits in the decision-tree models. Future research should validate these findings in prospective cohorts, incorporate objective activity measures (e.g., accelerometers), and include symptom burden, performance status, and treatment toxicity in predictive models. On the contrary, the strengths of this study include the large, heterogeneous sample and the application of an interpretable machine learning approach. Moreover, the separate examination of aerobic and resistance activities represents a step forward in identifying patients at risk of insufficient physical activity and in providing more appropriate and targeted support.

In conclusion, the decision-tree approach revealed that physical activity behaviors among patients with cancer are primarily driven by cancer type and sociodemographic characteristics. The low proportion of patients adhering to aerobic and resistance exercise guidelines underscores an urgent, largely unmet need for targeted physical activity interventions in oncology care. Importantly, recognizing that distinct determinants govern aerobic and resistance activities provides a critical framework for designing more precise, modality-specific exercise strategies that align with contemporary guidelines for patients with cancer.

## Data Availability

The datasets generated during and/or analysed during the current study are available from the corresponding author on reasonable request.
